# Integrative Analysis of Minichromosome Maintenance Proteins and Their Prognostic Significance in Melanoma

**DOI:** 10.3389/fonc.2021.715173

**Published:** 2021-08-19

**Authors:** Wei Han, Yi-Zhu Wu, Xiao-Yu Zhao, Zhen-Hua Gong, Guo-Liang Shen

**Affiliations:** ^1^Department of Burn and Plastic Surgery, The First Affiliated Hospital of Soochow University, Suzhou, China; ^2^Department of Surgery, Soochow University, Suzhou, China; ^3^Department of Burn and Plastic Surgery, Affiliated Hospital 2 of Nantong University, The First People’s Hospital of Nantong, Nantong, China

**Keywords:** melanoma, minichromosome maintenance, prognosis, TCGA, bioinformatics

## Abstract

**Background:**

Minichromosome maintenance (*MCM*) is known for participating in cell cycle progression, as well as DNA replication. While the diverse expression patterns and prognostic values of *MCM*s in melanoma still remained unclear.

**Methods:**

In the present study, the transcriptional and clinical profiles of *MCM*s were explored in patients with melanoma from multiple databases, including GEO, TCGA, ONCOMINE, GEPIA, UALCAN, cBioPortal, and TIMER databases.

**Results:**

We found that the elevated expressions of *MCM2–6* and *MCM10* were significantly expressed in melanoma compared to normal skin. High mRNA levels of *MCM4*, *MCM5*, and *MCM10* were closely related to worse prognosis in patients with melanoma. GSEA showed hallmark pathways were most involved in mTORC1 signaling, G2M checkpoint, E2F targets, and mitotic spindle. Furthermore, we found potential correlations between the *MCM* expression and the immune cell infiltration, including B cells, CD4+ T cells, CD8+ T cells, neutrophils, macrophages, and dendritic cells.

**Conclusion:**

Upregulated *MCM* gene expression in melanoma probably played a crucial part in the development and progression of melanoma. The upregulated *MCM4/5/10* expressions could be used as potential prognostic markers to improve the poor outcome and prognostic accuracy in patients with melanoma. Our study might shed light on the selection of prognostic biomarkers as well as the underlying molecular pathogenesis of melanoma.

## Introduction

Skin cutaneous melanoma (SKCM) is one of the most life-threatening skin malignancies. It accounts for approximately 80% of yearly deaths among patients with skin cancers worldwide (1). Formation of banal nevi, dysplastic nevi, melanoma *in situ*, and invasive melanoma are the four steps of the progression from melanocytes to SKCM (2). Surgical resection is the preferred treatment for primary melanoma, and metastatic melanoma is not sensitive to chemotherapy or radiotherapy (3). Immunotherapy has greatly improved in recent years and has been used to treat patients with SKCM. For example, immune checkpoint inhibitors that target programmed death ligand 1 (PD-L1) have been used, but such treatments benefit only a small number of patients with SKCM (4, 5). Therefore, early diagnosis of melanoma and identification of biomarkers are essential for effective and rapid intervention and treatment of patients with melanoma.

Genes that encode minichromosome maintenance proteins (*MCM*s) were originally found in *Saccharomyces cerevisiae*, which could be used to detect proteins that participate in plasmid maintenance in the cell cycle process (6). *MCM2–10* are highly conserved members of the *MCM* family. *MCM*s are crucial participants in the initiation and elongation of DNA replication and are considered to be potential indicators of cell proliferation (7). Because of their significant roles in DNA replication, *MCM*s are useful tools for diagnosis and prognosis prediction of tumors (8). *MCM1* (also known as *SRF*) is a transcription factor that is also required for minichromosome maintenance (9). Some members of the *MCM* gene family have been shown to be aberrantly highly expressed in multiple human malignancies, and upregulation of these genes contributes to tumor cell progression and prognosis prediction in patients with tumors (8, 10). However, comprehensive analysis of the clinical characteristics of *MCM*s in patients with SKCM has rarely been reported, and further exploration of their characteristics is still needed. In this study, we performed bioinformatics analysis to explore the expression patterns and prognostic significance of *MCM*s in patients with SKCM.

## Methods

### Gene Expression Profiling Interactive Analysis (GEPIA) Database

GEPIA (http://gepia.cancer-pku.cn/) could provide users with customizable and quick functionalities based on data from the Cancer Genome Atlas (TCGA; https://tcga-data.nci.nih.gov/tcga/) and the Genotype-Tissue Expression project (GTEx; https://www.gtexportal.org/home/index.html) (11). One-way ANOVA is applied when differential analysis is performed. Tumor stage or disease state is used as variables to calculate differential expressions. In addition, survival analyses based on gene expression levels can also be performed using a log-rank test by using the GEPIA. In our study, GEPIA is used to show the differential expressions and survival analyses of *MCM*s graphically among all TCGA cancers, as well as the correlations between tumor stages of SKCM patients and the expressions of *MCM*s.

### Oncomine Database

Oncomine online database (http://www.oncomine.com) is used to investigate the mRNA expressions of *MCM*s among 20 common tumors (12). Differential expressions of *MCM*s were selected by using the cut-off criteria: p = 0.01 (Student’s t-test), fold change = 1.5, and differentially expressed gene rank ≤10%.

### UALCAN

To analyze *MCM* expression in SKCM patients with different sample types, further UALCAN database analyses were performed as well (13). In the UALCAN analysis page, the TCGA dataset was defined as “skin cutaneous melanoma” for *MCM* expression analysis. The *MCM* expressions based on different sample types were analyzed.

### Statistical Analysis

Next, GraphPad Prism (version 8.0) was used to analyze and show the phenotype and transcriptional expression profiles in SKCM patients. The T1-2 and T3-4 among *MCM*s in melanoma from TCGA patients were compared, and p < 0.05 was considered of statistical significance.

### The Human Protein Atlas Database

The Human Protein Atlas database (https://www.proteinatlas.org/) is used to explore the immunohistochemistry (IHC)‐based protein expression profiles in normal tissues, different neoplasms, and cell lines (14). *MCM* protein expression IHC images in normal skin tissues as well as in clinical specimens of SKCM patients were downloaded from this database.

### cBioPortal for Cancer Genomics Dataset

The cBioPortal (http://cbioportal.org) could help users search for multidimensional cancer genomics datasets and obtain the data for over 5,000 tumor samples from more than 20 cancer studies (15). Thus, we used cBioPortal database to analyze *MCM* mutations in SKCM. In the current study, we analyzed the genomic alteration types and alteration frequency in SKCM by using cBioPortal, including mRNA upregulation, copy number amplification, deep deletion, and missense mutation with unknown significance.

### STRING Database

In this study, we used STRING (http://string-db.org; version 11.0) to show the protein co-regulation of *MCM*s and describe functional interactions among nodes (16). The interaction specificity score >0.4 (the default threshold in the STRING database) was considered statistically significant.

### The Database for Annotation, Visualization and Integrated Discovery (DAVID)

DAVID (https://david.ncifcrf.gov/) is an efficient online tool that can provide users with systematic and integrative functional annotations and help them explore biological meanings of target genes. Thus, it was applied to perform functional annotations and pathway enrichment analyses in this study. The biological process (BP), cellular component (CC), and molecular function (MF) are included in the gene ontology (GO) analyses, and Kyoto Encyclopedia of Genes and Genomes (KEGG) pathway enrichment analyses were performed for *MCM*s by DAVID (17, 18), visualized in the bubble charts. P-value <0.05 was considered of statistical significance.

### Cytoscape Software

ClueGO (version 2.5.3) of Cytoscape is a plug-in to analyze the GO and KEGG functional enrichment based on target genes (19). Thus, ClueGO was used to show and plot the GO (BP, CC, MF) and KEGG analyses.

### Gene Set Enrichment Analysis (GSEA)

GSEA tool (version 2.10.1) is a useful tool that can be utilized to detect the up- and downregulated genes, as well as the significantly changed pathways according to the expression data from the TCGA database (20). Student’s t-test statistical score is conducted in consistent pathways, and the mean of the differentially expressed genes is calculated in each separate analysis. The significantly involved hallmark pathways was identified by using a permutation test with 1,000 times. The adj. P using Benjamini and Hochberg (BH) and false discovery rate (FDR) method by default is applied to correct for the occurrence of false-positive results. An adj. P < 0.01 and FDR < 0.25 are considered to define the significantly involved genes.

### Tumor Immune Estimation Resource (TIMER) Database

TIMER (https://cistrome.shinyapps.io/timer/) is a straightforward website that provides a systematical analysis of immune infiltrates in various tumors (21). TIMER algorithm can estimate the abundances of six immune infiltrate (B cells, CD8+ T cells, CD4+ T cells, Neutrophils, Macrophages, and Dendritic cells). In this study, TIMER was conducted to perform a comprehensive correlation analysis between *MCM*s and tumor-infiltrating immune cell signatures.

## Results

### Expression Pattern and Survival Analysis of *MCM*s in Pan-Cancer Perspective

**Figure 1:**
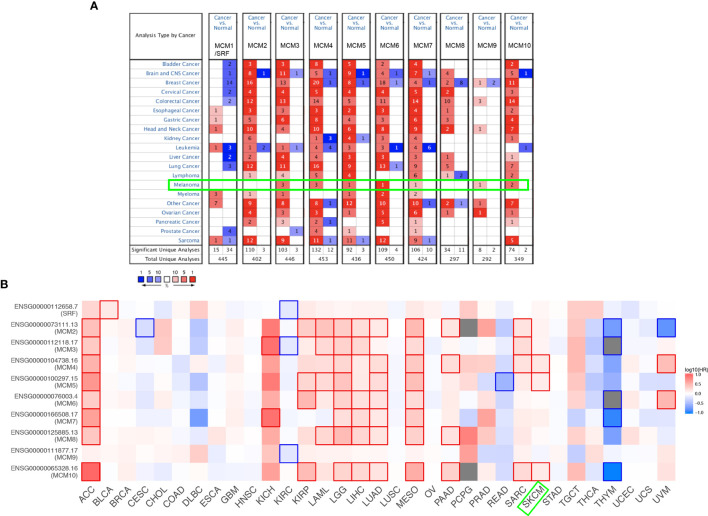
Expression level and survival analysis of *MCM*s by ONCOMINE and GEPIA. **(A)** The figure shows the numbers of datasets with statistically significant mRNA overexpression (red) or downregulated expression (blue) of *MCM*s. **(B)** Summary of hazard ratios (HR) illustrating cancer-*MCM* pairs with altered prognosis. ACC, Adrenocortical Carcinoma; BLCA, Bladder Urothelial Carcinoma; BRCA, Breast Invasive Carcinoma; CESC, Cervical Squamous Cell Carcinoma and Endocervical Adenocarcinoma; CHOL, Cholangiocarcinoma; COAD, Colon Adenocarcinoma; DLBC, Lymphoid Neoplasm Diffuse Large B-cell Lymphoma; ESCA, Esophageal Carcinoma; HNSC, Head and Neck Squamous Cell Carcinoma; KICH, Kidney Chromophobe; KIRC, Kidney Renal Clear Cell Carcinoma; KIRP, Kidney Renal Papillary Cell Carcinoma; LAML, Acute Myeloid Leukemia; LGG, Brain Lower Grade Glioma; LIHC, Liver Hepatocellular Carcinoma; LUAD, Lung Adenocarcinoma; LUSC, Lung Squamous Cell Carcinoma; MESO, Mesothelioma; OV, Ovarian Serous Cystadenocarcinoma; PAAD, Pancreatic Adenocarcinoma; PCPG, Pheochromocytoma and Paraganglioma; PRAD, Prostate Adenocarcinoma; READ, Rectum Adenocarcinoma; SARC, Sarcoma; SKCM, Skin Cutaneous Melanoma; STAD, Stomach Adenocarcinoma; TGCT, Testicular Germ Cell Tumors; THCA, Thyroid carcinoma; THYM, Thymoma; UCEC, Uterine Corpus Endometrial Carcinoma; UCS, Uterine Carcinosarcoma; UVM, Uveal Melanoma.

As shown in **Figure 1A**, *MCM*s were mostly overexpressed in many cancer types by using the Oncomine database. In melanoma, upregulation of all *MCM*s was observed in cancer tissues, except *MCM1/2/8*, which may be due to the limited samples. Transcriptional expression of *MCM*s in SKCM is analyzed and displayed in **Table 1**. *MCM3* and *MCM4* overexpression were present in Talantov’s datasets (22) and Haqq’s datasets (23). In Riker’s dataset, the transcription levels of *MCM5* and *MCM6* in melanoma are higher than those in normal skin tissues, and those fold changes are 2.119 and 2.392 (24). *MCM7* is significantly upregulated in benign melanocytic skin nevus, with fold changes of 8.786 in Talantov’s dataset (22). The overexpression of *MCM9* and *MCM10* was observed in Haqq’s datasets (23) and Talantov’s datasets (22), respectively. Then, Kaplan-Meier curve, as well as log-rank test analyses, weas applied to show the overall survival (OS). Results are graphically demonstrated in **Figure 1B**, and there was an apparent heterogeneity between different types of tumors. Three members of *MCM*s were greatly related to OS in SKCM patients.

**Table 1:** Comparison of mRNA expression of *MCM*s in melanoma and normal skin tissues from the Oncomine database.

	Type	Fold Change	P value	t-test	Ref.
*MCM3*					
	Cutaneous Melanoma *vs.* Normal	2.355	6.73E-08	11.571	Talantov Melanoma
	Cutaneous Melanoma *vs.* Normal	3.698	0.000229	6.615	Haqq Melanoma
	Non-Neoplastic Nevus *vs.* Normal	1.975	0.000839	4.256	Haqq Melanoma
*MCM4*					
	Cutaneous Melanoma *vs.* Normal	5.476	0.000181	7.137	Haqq Melanoma
	Cutaneous Melanoma *vs.* Normal	9.864	1.75E-07	13.419	Talantov Melanoma
	Benign Melanocytic Skin Nevus *vs.* Normal	3.096	0.000141	4.307	Talantov Melanoma
*MCM5*					
	Cutaneous Melanoma *vs.* Normal	2.199	0.000502	4.109	Riker Melanoma
*MCM6*					
	Cutaneous Melanoma *vs.* Normal	2.392	0.0000269	5.662	Riker Melanoma
*MCM7*					
	Benign Melanocytic Skin Nevus *vs.* Normal	8.786	0.000195	4.858	Talantov Melanoma
*MCM9*					
	Cutaneous Melanoma *vs.* Normal	2.149	0.001	4.864	Haqq Melanoma
*MCM10*					
	Cutaneous Melanoma *vs.* Normal	2.721	0.0000899	5.101	Talantov Melanoma
	Benign Melanocytic Skin Nevus *vs.* Normal	3.361	0.0000198	5.451	Talantov Melanoma

### Transcriptional Levels of *MCM*s in Patients With SKCM

**Figure 2:**
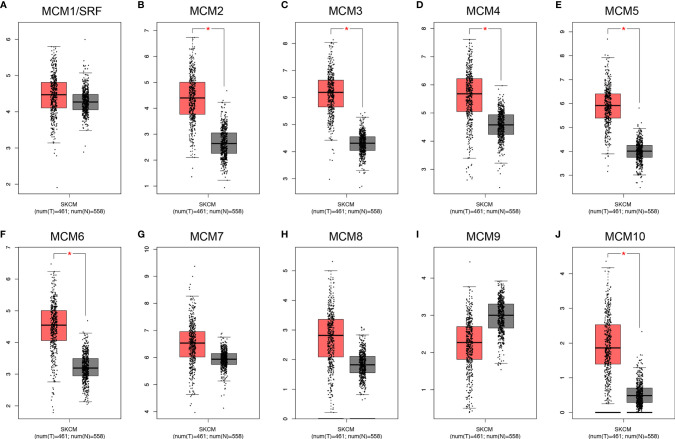
Transcriptional expression of distinct *MCM* family members in TCGA cohort. **(A–J)** Except for *MCM9*, GEPIA analyses showed that *MCM1–8* and *MCM10* were overexpressed in SKCM than in normal skin, although statistically significant differences were observed for *MCM2–6* and *MCM10* only (*p < 0.05).

**Figure 3:**
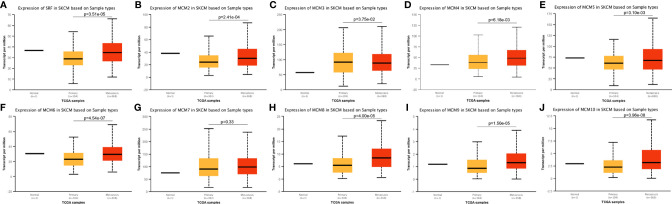
Transcriptional expression of *MCM*s in different sample types of melanoma. **(A–J)** Except *MCM7*, *MCM1–6* and *MCM8–10* were all overexpressed in metastatic melanoma than in primary melanoma in the TCGA cohort (*p* < 0.05).

Except for *MCM9*, GEPIA analyses showed that *MCM1-8* and *MCM10* were overexpressed in SKCM than in normal skin (**Figure 2**), although statistically significant differences were observed for *MCM2–6* and *MCM10* only. Furthermore, the expression level of *MCM*s between primary and metastatic melanoma was also compared in this study. Except for *MCM7*, *MCM1–6* and *MCM8–10* were all highly expressed in metastatic melanoma than in primary melanoma in the TCGA cohort (P < 0.05) (**Figure 3**).

### Relationship Between the Clinicopathological Parameters of SKCM Patients and the mRNA Levels of *MCM*s

**Figure 4:**
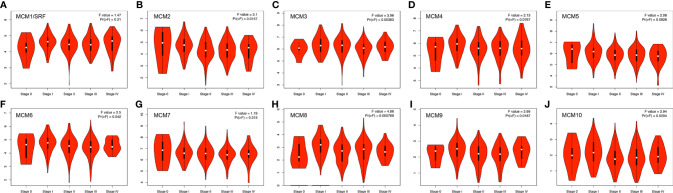
**(A–J)** Correlation between *MCM* expression and tumor stage in SKCM patients (GEPIA). *MCM2*, *MCM3*, *MCM6*, *MCM8*, *MCM9*, and *MCM10* groups significantly varied and associated with the tumor stages.

**Figure 5:**
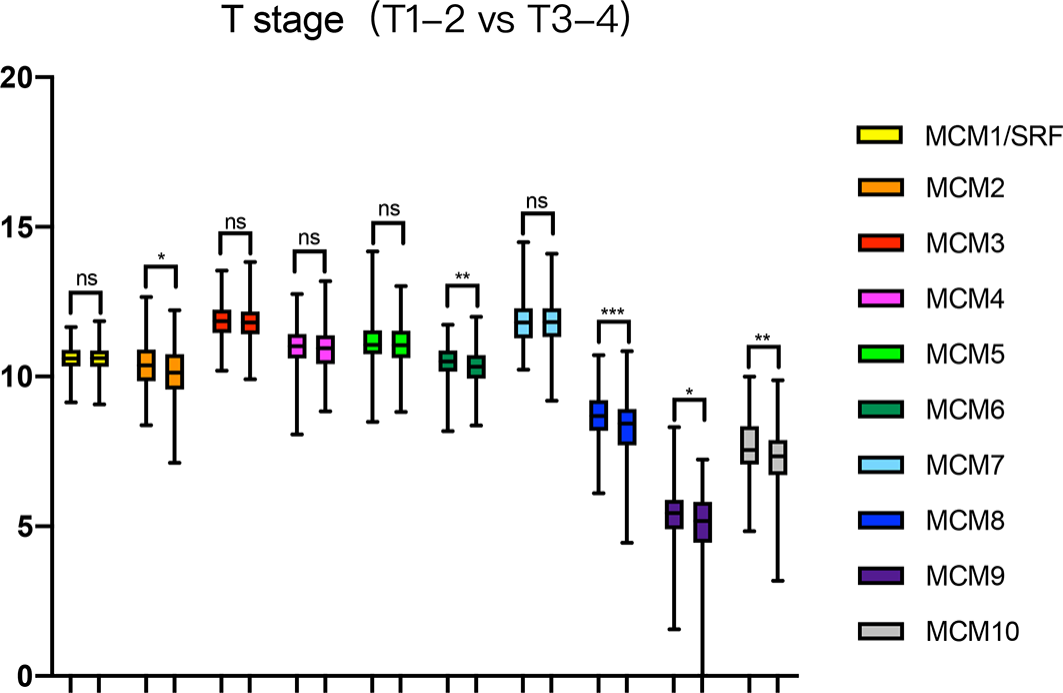
Relationship between transcriptional expressions of distinct *MCM* family members and T stage of SKCM patients. *MCM2/6/8/9/10* showed significant correlations with T stages in SKCM patients (ns, no significance; *p < 0.05, **p < 0.01, ***p < 0.001).

Next, we analyzed the expressions of *MCM*s with tumor stages for SKCM by GEPIA. *MCM2*, *MCM3*, *MCM6*, *MCM8*, *MCM9*, and *MCM10* groups significantly varied and associated with the tumor stages (**Figure 4**). In addition, expression patterns of *MCM2*, *MCM6*, *MCM8*, *MCM9*, and *MCM10* were greatly related to the T stage (T1-T2 *vs* T3-T4) (**Figure 5**).

### Relationship Between the Survival Outcomes of SKCM Patients and the mRNA Levels of *MCM*s

**Figure 6:**
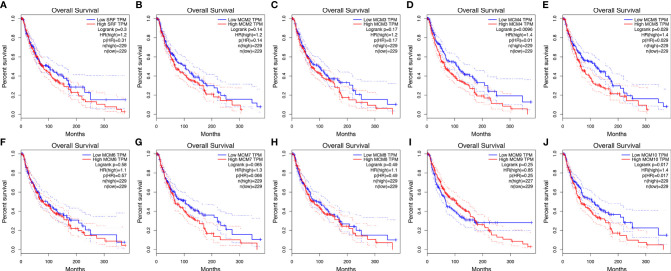
**(A–J)** Kaplan-Meier survival analyses on differential *MCM* expression groups with OS in the TCGA SKCM patients. High expression of *MCM4/5/10* were significantly correlated with poor OS.

Then, GEPIA was applied to show the prognostic value of *MCM*s in SKCM patients, as well as the correlation between survival status of SKCM patients and *MCM* mRNA expression. Results indicated that the higher expressions of *MCM4*, *MCM5*, and *MCM10* were significantly associated with the worse OS in SKCM patients (**Figure 6**).

### Protein Expression Levels of *MCM*s in Patients With SKCM

**Figure 7:**
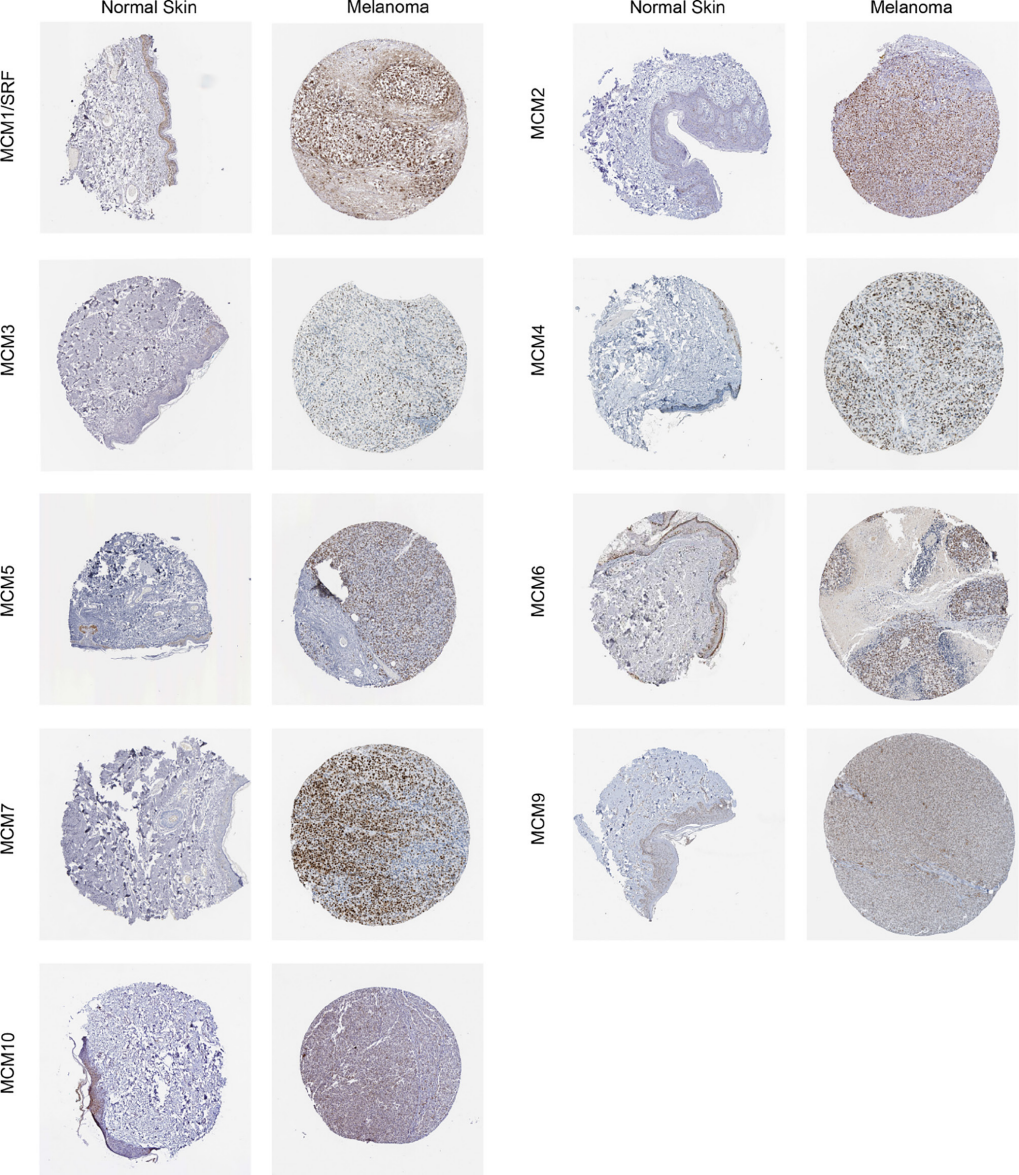
Immunohistochemical staining for protein expression of *MCM*s in tissues from patients with SKCM and normal tissues. The results revealed *MCM1–7* protein levels were higher in melanoma tissues than in normal skin tissues, consistent with the results of *MCM* mRNA expression, whereas *MCM9* and *MCM10* proteins showed no great difference between melanoma and normal skin.

Furthermore, IHC staining images for the *MCM* proteins in both normal skin tissues and melanoma were downloaded from the Human Protein Atlas database to investigate the differentially expressed *MCM* proteins in melanoma tissues (**Figure 7**). The results revealed *MCM1–7* protein levels were higher in melanoma tissues than in normal skin tissues, consistent with the results of *MCM* mRNA expression, whereas *MCM9* and *MCM10* proteins showed no great difference between melanoma and normal skin.

### Genetic Mutations of *MCM*s in SKCM

**Figure 8:**
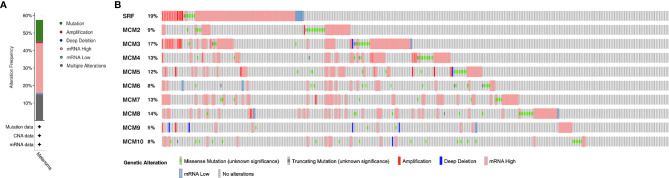
Genetic mutations in *MCM* family members (cBioPortal). **(A)** A visual summary of alteration based on a query of 10 *MCM*s, which was altered in 255 samples out of 444 SKCM patients (57.43%). **(B)** *MCM1* (SRF) (19%) was the most frequently altered gene among the *MCM* genes, followed by *MCM3* (17%), *MCM4* (13%), and *MCM7* (13%), including amplification, deep deletion, and missense mutations.

*MCM* alterations and correlations were analyzed by cBioPortal for TCGA SKCM cohort. *MCM*s were altered in 255 samples out of 444 SKCM patients (57.43%). *MCM1(SRF)* (19%) was the most frequently altered gene among the *MCM* genes, followed by *MCM3* (17%), *MCM4* (13%), and *MCM7* (13%), including amplification, deep deletion, and missense mutations (**Figure 8**).

### Predicted Interaction Networks and Signaling Pathways of *MCM*s

**Figure 9:**
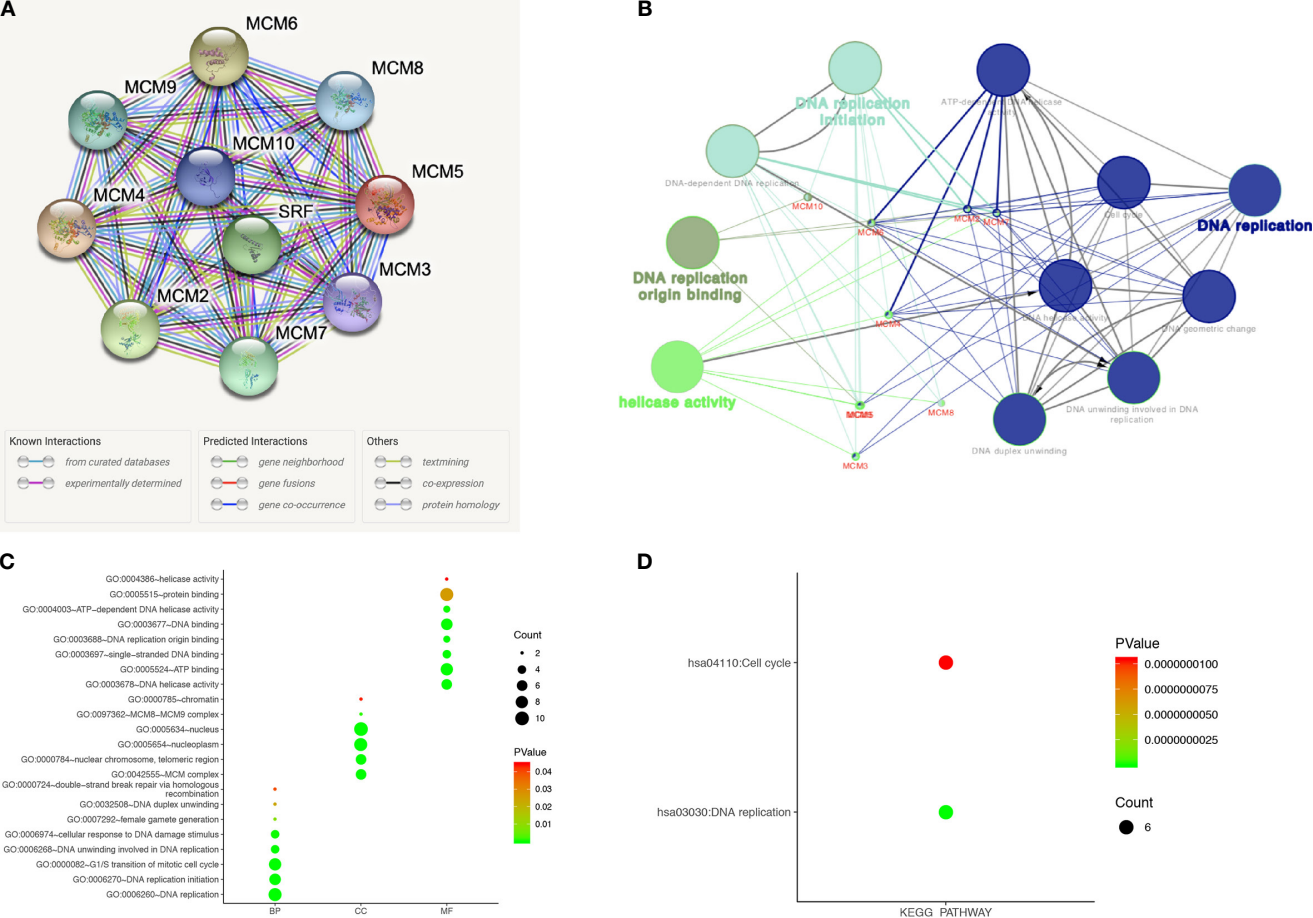
Functions enrichment and signaling pathways analysis of the mutations in *MCM*s in SKCM patients. **(A)** A PPI network analysis of differentially expressed *MCM*s with STRING was conducted to show the close interactions among them. **(B)** The functional annotation analyses of *MCM*s were constructed using ClueGO, including DNA replication, DNA replication initiation, helicase activity, and DNA replication origin binding. (**C, D**) GO and KEGG pathway enrichment analyses of *MCM*s were performed using DAVID and visualized in bubble chart. The biological processes of *MCM*s were greatly enriched in DNA replication, G1/S transition of the mitotic cell cycle, DNA replication initiation, DNA unwinding involved in DNA replication, cellular response to DNA damage stimulus, and DNA duplex unwinding. Changes in cellular components were significantly enriched in the nucleus, nucleoplasm, nuclear chromosome, telomeric region, and MCM complex. As for molecular function, changes were mostly enriched in protein binding, ATP binding, DNA binding, DNA helicase activity, and single-stranded DNA binding. KEGG analyses revealed that *MCM*s were most involved in cell cycle and DNA replication (BP, biological process; CC, cellular component; MF, molecular function).

A protein-protein interaction (PPI) network of differentially expressed *MCM*s was conducted to identify the potential interactions among them by STRING (**Figure 9A**). Then, ClueGO functional annotations performed a network of the *MCM* genes (**Figure 9B**). The GO and KEGG enrichment analyses of the *MCM*s are presented in different colors (**Figure 9B**). The detailed functional notes and classification pie charts are listed in **Supplementary Figure 1**; 63.64% of terms belong to DNA replication, 18.18% to DNA replication initiation, 9.09% to helicase activity, and 9.09% to DNA replication origin binding.

The functions of *MCM*s were also predicted by analyzing GO in the DAVID, visualized in bubble charts (**Figure 9C**). The biological processes of *MCM*s were greatly enriched in DNA replication, G1/S transition of the mitotic cell cycle, DNA replication initiation, DNA unwinding involved in DNA replication, cellular response to DNA damage stimulus, and DNA duplex unwinding. Changes in cellular components were significantly enriched in the nucleus, nucleoplasm, nuclear chromosome, telomeric region, and *MCM* complex. As for molecular function, changes were mostly enriched in protein binding, ATP binding, DNA binding, DNA helicase activity, and single-stranded DNA binding. KEGG analyses revealed that *MCM*s were most involved in the cell cycle and DNA replication (**Figure 9D**).

**Figure 10:**
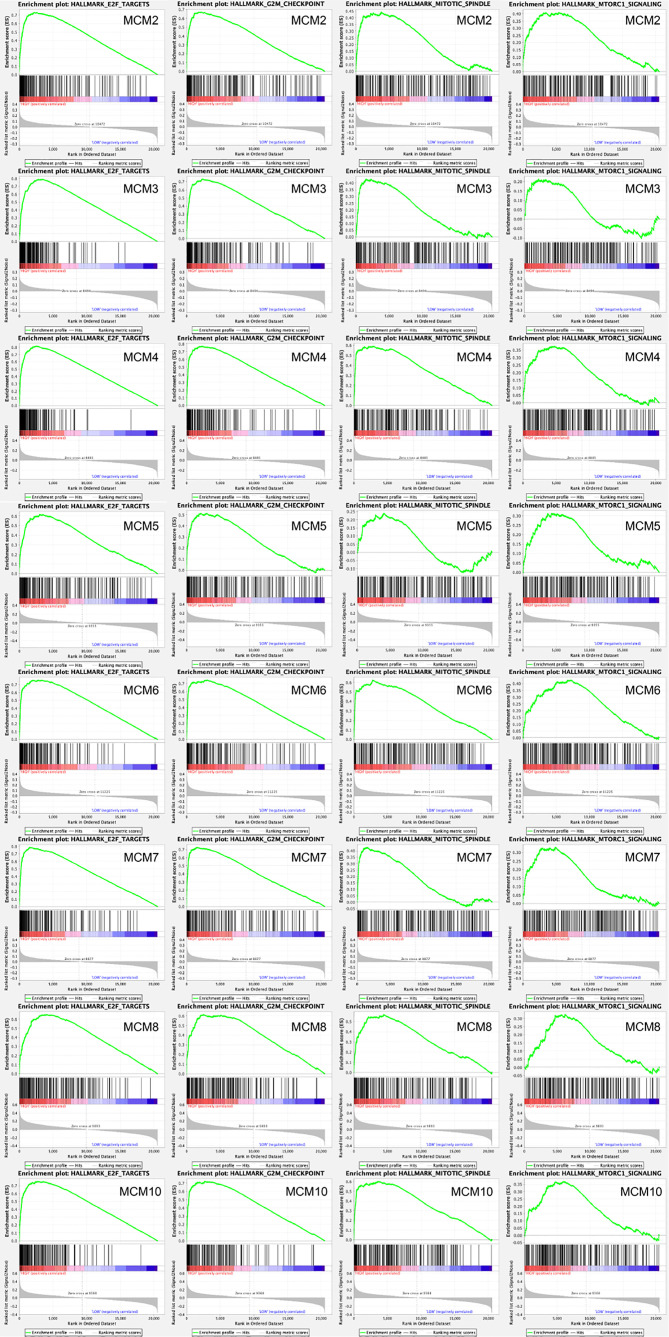
GSEA was used to perform hallmark signaling analysis in *MCM*s, respectively. A total of 100 significant genes were obtained from GSEA with positive and negative correlation. GSEA, including *MCM2–8* and *MCM10*, indicated that the most involved hallmark pathways were E2F targets, G2M checkpoint, mitotic spindle, and mTORC1 signaling.

Afterwards, a total of 100 significantly changed genes with positive correlation obtained from GSEA were plotted. GSEA, including *MCM2–8* and *MCM10*, showed that the most involved hallmark pathways were E2F targets, G2M checkpoint, mitotic spindle, and mTORC1 signaling. The details are displayed in **Figure 10**.

### Immune Cell Infiltration of *MCM*s in Patients With SKCM

**Figure 11:**
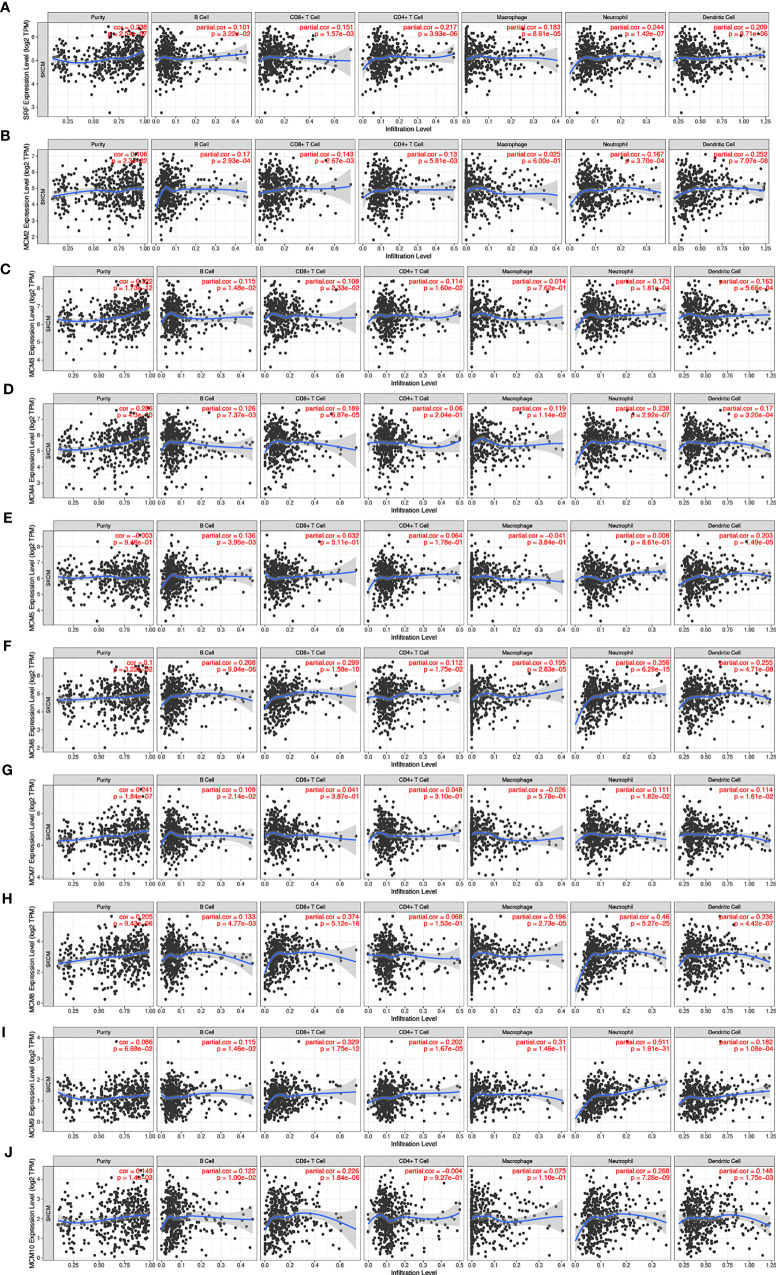
**(A–J)** The correlation between different expressed *MCM*s and immune cell infiltration (TIMER). There was a positive correlation between *MCM1/2/3/7/8/10* expression and the infiltration of immune cells. Instead, *MCM4/5/6/9* were negatively associated with the infiltration of immune cells.

Furthermore, we explored the correlations between differentially expressed *MCM*s and immune cell infiltration by TIMER database. There was a positive correlation between *MCM1* expression and the infiltration of B cells (Cor = 0.101, *p* = 3.22e−2), CD8+T cells (Cor = 0.151, *p* = 1.57e−3), CD4+T cells (Cor = 0.217, *p* = 3.93e−6), macrophages (Cor = 0.183, *p* = 8.61e−5), neutrophils (Cor = 0.244, *p* = 1.42e−7), and dendritic cells (Cor = 0.209, *p* = 8.71e−6; **Figure 11A**). Similar results were observed for *MCM2* and *MCM3*. *MCM2* and *MCM3* expressions were positively associated with the infiltration of B cells, CD8+T cells, CD4+T cells, neutrophils and dendritic cells (**Figures 11B, C**). The expression of *MCM4* was negatively associated with the infiltration of B cells (Cor = 0.126, *p* = 7.73e−5), CD8+ T cells (Cor = 0.189, *p* = 6.87e−5), macrophages (Cor = 0.119, *p* = 1.14e−2), neutrophils (Cor = 0.238, *p* = 2.92e−7), and dendritic cells (Cor = 0.17, *p* = 3.20e−4; **Figure 11D**). There was a negative correlation between *MCM5* expression and the infiltration of B cells (Cor = 0.136, *p* = 3.95e−3) and dendritic cells (Cor = 0.203, *p* = 1.49e−5; **Figure 11E**). *MCM6* and *MCM9* expressions negatively correlated with infiltration of the all six immune cell types (B cells, CD8+ T cells, CD4+ T cells, macrophages, neutrophils, and dendritic cells; all *p* < 0.05; **Figures 11F, I**). Similarly, the expressions of *MCM7* and *MCM10* were also positively associated with the infiltration of B cells, neutrophils and dendritic cells (**Figures 11G, J**).We also found that the higher the infiltrations of B cells (Cor = 0.133, *p* = 4.77e−3), CD8+ T cells (Cor = 0.374, *p* = 5.12e−16), macrophages (Cor = 0.196, *p* = 2.73e−5), neutrophils (Cor = 0.46, *p* = 5.27e−25), and dendritic cells (Cor = 0.236, *p* = 4.42e−7), the higher the expressions of *MCM8* (**Figure 11H**).

## Discussion

*MCM*s have recently been found to be effective biomarkers for the diagnosis and prognosis prediction of various tumors. *MCM*s can be used to distinguish differentiated cells from undifferentiated cells because of their decreased abundance following cell cycle exit (25). One significant hallmark of tumors is the loss of differentiation and unscheduled re-entry into the cell cycle (7). However, there are few reports about the functions of *MCM*s in melanoma. In this study, we found that that *MCM*s were heterogeneous in different types of tumors. According to the pan-cancer expression profiling analysis and survival analysis of TCGA data, we identified three *MCM* genes that were significantly associated with the prognosis of patients with SKCM. We also found that *MCM* mRNA levels were correlated with the T and tumor stages of SKCM. The GO and KEGG functional analyses indicated that the *MCM* genes that were differentially expressed between melanoma and normal skin tissues were significantly enriched in cell cycle and DNA replication. The GSEA showed that the most involved hallmark pathways were E2F targets, G2M checkpoint, mitotic spindle, and mTORC1 signaling. It has been shown previously that mTORC1 signaling was regulated by both MAPK and PI3K signaling and played an essential role in melanoma cell proliferation (26). Furthermore, the proliferation of T lymphocytes were induced by IL-4 with the coordinate transcriptional induction of the cell cycle regulatory genes that encode *MCM*s (27). Therefore, we investigated the correlation between differentially expressed *MCM* genes and immune cell infiltration using the TIMER database and showed that *MCM*s may be involved in immune responses and thus might affect the clinical outcome of patients with melanoma. These results demonstrate that differentially expressed *MCM* genes may play key roles in SKCM.

Altered *MCM2* expression was shown to signify cell-cycle deregulation, which is necessary for the initiation and progression of cancers (28). Increased expression of *MCM2* was found to be an independent adverse prognostic factor, and *MCM2* has served as a prognostic marker for multiple myeloma (29). *MCM2* was shown to be a more sensitive proliferation biomarker than Ki67 and geminin in oral melanoma (30). *MCM2* was also found to be associated with lower survival rates, suggesting its possible role as a prognostic predictor in melanoma (31). The different expression levels of *MCM2* in melanocytic neoplasms potentially provide a useful tool to distinguish benign melanocytic lesions from malignant melanocytic lesions (32). In our study, the GEPIA showed that the *MCM2* mRNA level was much higher in melanoma than in normal skin tissues. Expression of *MCM2* was significantly associated with the clinical characteristics (e.g., T stage and tumor stage) of patients with SKCM. Although the statistical significance was low, we found that high *MCM2* expression was associated with poor OS in patients with melanoma, which is consistent with the results of the previous study (31).

*MCM3* was found to be more reliable prognosis biomarker than Ki-67 for malignant melanoma and salivary gland tumors (33). Nodin et al. (34) reported the *MCM3* expression could be considered as an independent prognostic biomarker for patients with primary melanoma, and the absence of *MCM3* was correlated with tumor progression and worse outcomes in SKCM. The expression of *MCM3* was also associated with poor survival outcomes in medulloblastoma and malignant glioma (35). In the present study, *MCM3* was found to be overexpressed in melanoma tissue compared with normal skin tissue. We also demonstrated that *MCM3* expression was significantly associated with the pathological stages in patients with SKCM. Similarly, we found elevated expression of *MCM3* was related to worse prognosis in melanoma patients, which is consistent with the previous study (34), but the statistical significance was low. The TIMER analysis showed that *MCM3* was closely correlated with B cells and CD4+ and CD8+ T cells, which may provide a new approach to immunotherapy for melanoma.

Upregulation of *MCM4* was found to be a potential prognostic biomarker for lung cancers by coordinating the cell cycle, DNA replication, and other biological processes and pathways (36). The loss of *MCM4* improved the therapeutic effect of cisplatin because of the induced DNA damage, indicating that *MCM4* could be a potential cancer treatment target in cervical cancer (33). Moreover, significantly more *MCM4* was expressed in melanoma compared with in-nevi tissue (37). Orange (38) demonstrated that a mutation in the *MCM4* gene could be a genetic cause of natural killer (NK) cell deficiency and suggested a critical role for *MCM*s in NK cells and in NK cell–mediated host defense. In this study, we found that the expression of *MCM4* was higher in melanoma than in normal skin tissues. Importantly, high expression of *MCM4* was found to be highly associated with worse OS in patients with melanoma, and *MCM4* had a positive correlation with B cells and T cells.

*MCM5* was overexpressed in lung cancers, and elevated *MCM5* expression was associated with increased morbidity (39). The *SOX10–MCM5* axis was found to be a significant pathway that regulates melanocyte proliferation, which is required for melanocyte survival (40). Knockdown of *SOX10* in melanocytes inhibited cell proliferation and decreased *MCM5* expression (40). In the present study, we found that *MCM5* was upregulated in melanoma compared with its expression in normal skin and that increased expression of *MCM5* was significantly associated with poor OS of patients with SKCM.

Overexpression of *MCM6* is related to the diagnosis and poor prognosis of hepatocellular carcinoma (41). *MCM6* has also been used to identify cancer cell proliferation and may be a useful prognostic biomarker in diffuse large B-cell lymphoma (42). However, few studies have reported its role in melanoma. In this study, we found that *MCM6* expression was higher in melanoma than in normal skin in our analysis of TCGA data, and it was closely associated with T and tumor stages in patients with melanoma.

*MCM7* was involved in oncogenic signaling pathways and was highly expressed in various tumor tissues, including melanoma. *MCM7* silencing promoted autophagy and apoptosis, which inhibited the migration, viability, and invasion of tumor cells in melanoma (43). *MCM7* was significantly correlated with tumorigenesis, progression, malignant conversion, and prognosis in skin squamous cell carcinoma (44). In our study, although it was not statistically significant, we found that *MCM7* was more highly expressed in melanoma than in normal skin, and increased expression of *MCM7* was correlated with worse outcomes in melanoma, which is consistent with its oncogenic role.

*MCM8* is involved in the repair of double-strand DNA breaks and homologous recombination (45). Loss of *MCM8* can induce a higher apoptotic rate as well as lower cell viability. *MCM8* was found to be helpful in treating patients with chronic myelogenous leukemia (29). In our study, high expression of *MCM8* was closely related to the T and tumor stages. *MCM8* also was associated with immune cell infiltration in patients with melanoma, which may provide novel insights for the diagnosis and treatment of malignant melanoma.

*MCM9* is found only in vertebrates and is more closely related to *MCM8* than to the other *MCM* family members, implying a recent duplication of *MCM8* (7). The relationship between *MCM9* and cancers remains elusive. *MCM9* knockout mice were more likely to have ovarian and liver tumors than wild-type mice (46). Moreover, carriers of homozygous mutations in *MCM9* had a high risk of early colorectal carcinoma and premature ovarian insufficiency (47). In our study, *MCM9* was correlated with the T and tumor stages in patients with melanoma. We noticed that the expression and survival curve of *MCM9* was quite different from those of other *MCM* family members. This finding needs to be studied further in the future.

*MCM10* is an essential protein in DNA replication by forming complexes with *MCM2–7*, and it participates in multiple molecular and cellular processes, such as DNA replication, early embryogenesis, and normal embryonic development. *MCM10* is also involved in the formation and development of multiple tumors, including glioma, prostate cancer, urothelial cancer, neuroblastoma, and breast cancer (48). Together with *MCM7*, the upregulation of *MCM10* expression was associated with an increase of NK cells (49). In the present study, we found that *MCM10* was overexpressed in melanoma compared with its expression in normal skin, and showed significant differences in expression at the T and pathological stages. Increased *MCM10* expression was closely associated with poor prognosis in patients with SKCM.

Although *MCM1* is not a member of the *MCM* family, it is also required for minichromosome maintenance and plays a role in DNA synthesis (7). MCM1 interacts with serum response elements to activate the immediate-early genes and further perform its function. Notably, *MCM1* is the downstream target of the mitogen-activated protein kinase (MAPK) pathway (50), which is the pathway most involved in SKCM, suggesting that *MCM1* may play a role in melanoma tumorigenesis.

This is the first study to systematically investigate the oncogenic and prognostic utility of *MCM*s in SKCM. Although the potential role of *MCM*s in various tumors has been reported previously, little was known about their involvement in SKCM. In this study, we found that *MCM*s were significantly overexpressed in SKCM and were associated with T stage, tumor stage, and OS. We also discovered that genetic mutations in *MCM*s were present in 57.43% of the patients with SKCM. Importantly, we identified significant correlations between the expression of *MCM*s and the infiltration of immune cells (B cells, CD4+ T cells, CD8+ T cells, macrophages, neutrophils, and dendritic cells), which may provide new clues for immunotherapeutic targets and prognostic markers for melanoma. However, further experiments are required to validate our findings and promote the clinical utility of *MCM*s as prognostic biomarkers or therapeutic targets for patients with melanoma.

In summary, we detected elevated expression levels of *MCM2–6* and *MCM10* in melanoma and found that increased *MCM4/5/10* mRNA levels were associated with a worse prognosis for patients with melanoma. In the future study, further functional works, as well as validated cohorts, are needed to verify the absoluteness of these findings, which will provide new insights into the molecular alterations in SKCM and will contribute to finding more potential biomarkers in this area.

## Data Availability Statement

The original contributions presented in the study are included in the article/**Supplementary Material**. Further inquiries can be directed to the corresponding authors.

## Author Contributions

G-LS, Z-HG, and X-YZ developed the idea, designed the research, and revised the writing. WH and Y-ZW analyzed the data and drafted the manuscript. All authors contributed to the article and approved the submitted version.

## Funding

Medical Research Project of Jiangsu Provincial Health Commission (Z2019031); Medical Research Project of Nantong Health Commission (MA2020001); Nantong Science and Technology Bureau (MS12021093, JCZ21126).

## Conflict of Interest

The authors declare that the research was conducted in the absence of any commercial or financial relationships that could be construed as a potential conflict of interest.

## Publisher’s Note

All claims expressed in this article are solely those of the authors and do not necessarily represent those of their affiliated organizations, or those of the publisher, the editors and the reviewers. Any product that may be evaluated in this article, or claim that may be made by its manufacturer, is not guaranteed or endorsed by the publisher.
