# Supplementary figures and images for: Integrative Analysis of Minichromosome Maintenance Proteins and Their Prognostic Significance in Melanoma
Source: Front Oncol. 2021 Aug 19;11:715173. doi: 10.3389/fonc.2021.715173 (PMC8417415; doi:10.3389/fonc.2021.715173)

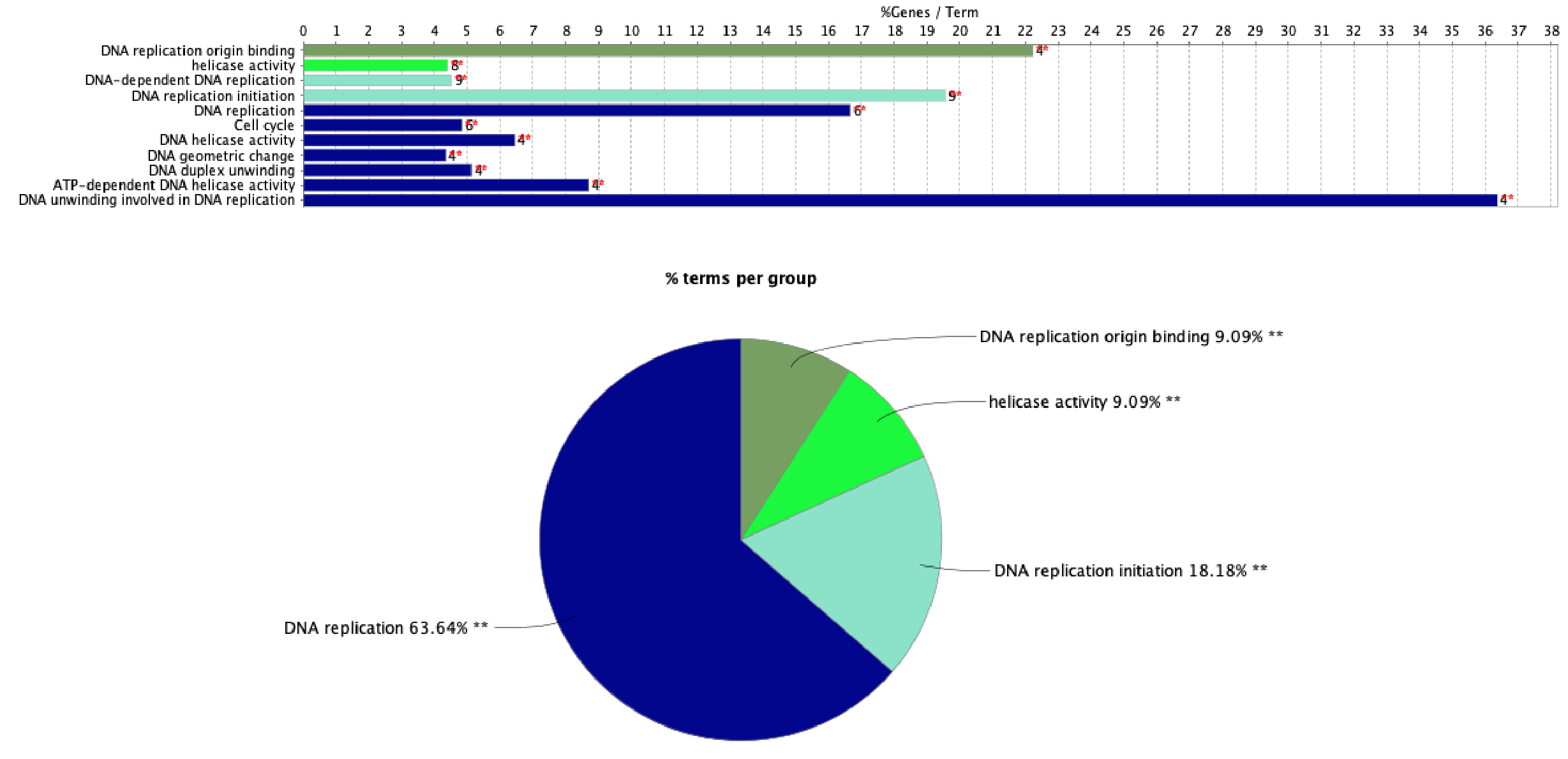

Supplement: Supplementary file 1 [file Image_1.tif]
